# Supplementary material for: New-Onset Paroxysmal Atrial Fibrillation in the Setting of Acute Pulmonary Embolism Is Associated with All-Cause Hospital Mortality in Women but Not in Men
Source: Diagnostics (Basel). 2023 May 23;13(11):1829. doi: 10.3390/diagnostics13111829 (PMC10252450; doi:10.3390/diagnostics13111829)
Supplement: Supplementary file 1 [file diagnostics-13-01829-s001.zip › diagnostics-2311341-supplementary.pdf]

Supplementary Table S1. Several hemodynamic, echocardiographic and biomarker parameters in women vs men who had paroxysmal AF and acute PE. Patients with EFLV<50% are included. Variables were presented as means±SD or medians with 25th-75th . Missing values were existing in 30-40% of echocardiographic variables (registry data).

Parameters measured during the first 24 hours from admission	Women N=68	Men N=58	p
HR – beat/min	117±33	115±23	0.625
SAP – mmHg	118±21	115±27	0.709
PO_2 – mmHg	62.22±21.41	63.71±20.64	0.739
RV-diameter (4-CH view) – mm	33.5 (28.0-40.0)	37.5 (33.2-42.8)	0.011
RV-diameter/ BSA – mm	18.7 (15.7-22.8)	18.5 (16.4-21.6)	0.636
RVSP – mmHg	50 (39-60)	50 (40-60)	0.977
TAPSE – mm	1.5 (1.3-2.0)	1.5 (1.2-2.0)	0.828
TAPSE/RVSP – mm/mmHg	0.028 (0.023-0.040)	0.027 (0.022-0.035)	0.679
RV/LV > 1.0	18/37 (32.7%)	21/46 (45.7%)	0.221
C-reactive protein – mg/dl	71.8 (20.4-127.5)	76.8 (28.0-161.2)	0.355
Total Leukocyte count x 10^9/l	11.9 (9.3-14.6)	11.3 (9.3-14.9)	0.925
Hb – g/l	124.5±21.5	135.6±23.7	0.562
BNP – pg/ml	369 (266-687)	212 (150-735)	0.071
cTnI – ng/ml	0.12 (0.03-0.34)	0.07 (0.02-0.57)	0.933

HR – heart rate, SAP – systolic arterial pressure, PO_2 partial oxygen concentration in arterial blood, RV – right ventricle, 4-CH – four chamber, RVSP – right ventricle systolic pressure, TAPSE – tricuspide annulus plane systolic excursion, LV – left ventricle, Hb – hemoglobin, BNP – brain natriuretic peptide, cTnI – cardiac troponin I.

Supplementary Figure S1. Cardiac troponin I blood levels at admission in patients with paroxysmal AF during acute PE with respect of sex. Boxes represents 25th-75th values with median line.

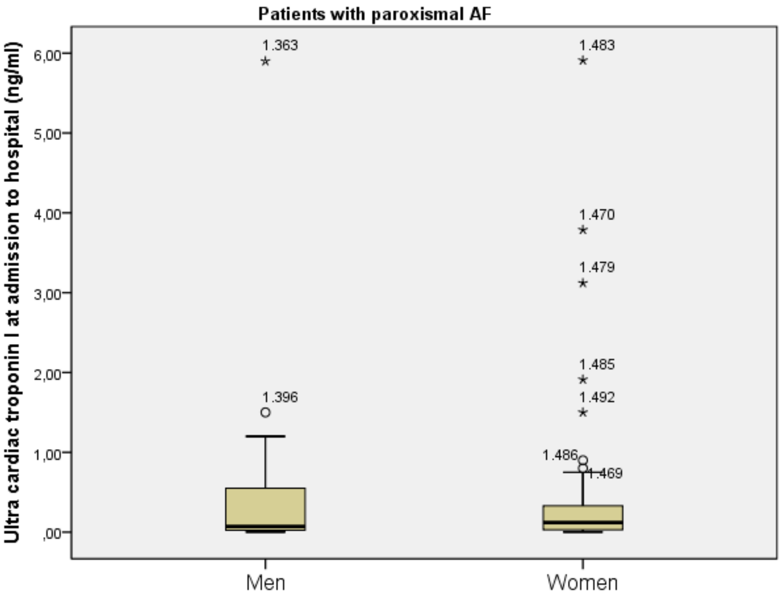

Figures S2. All-cause death regarding the presence of new onset paroxysmal AF in women vs men across the mortality risk stratums

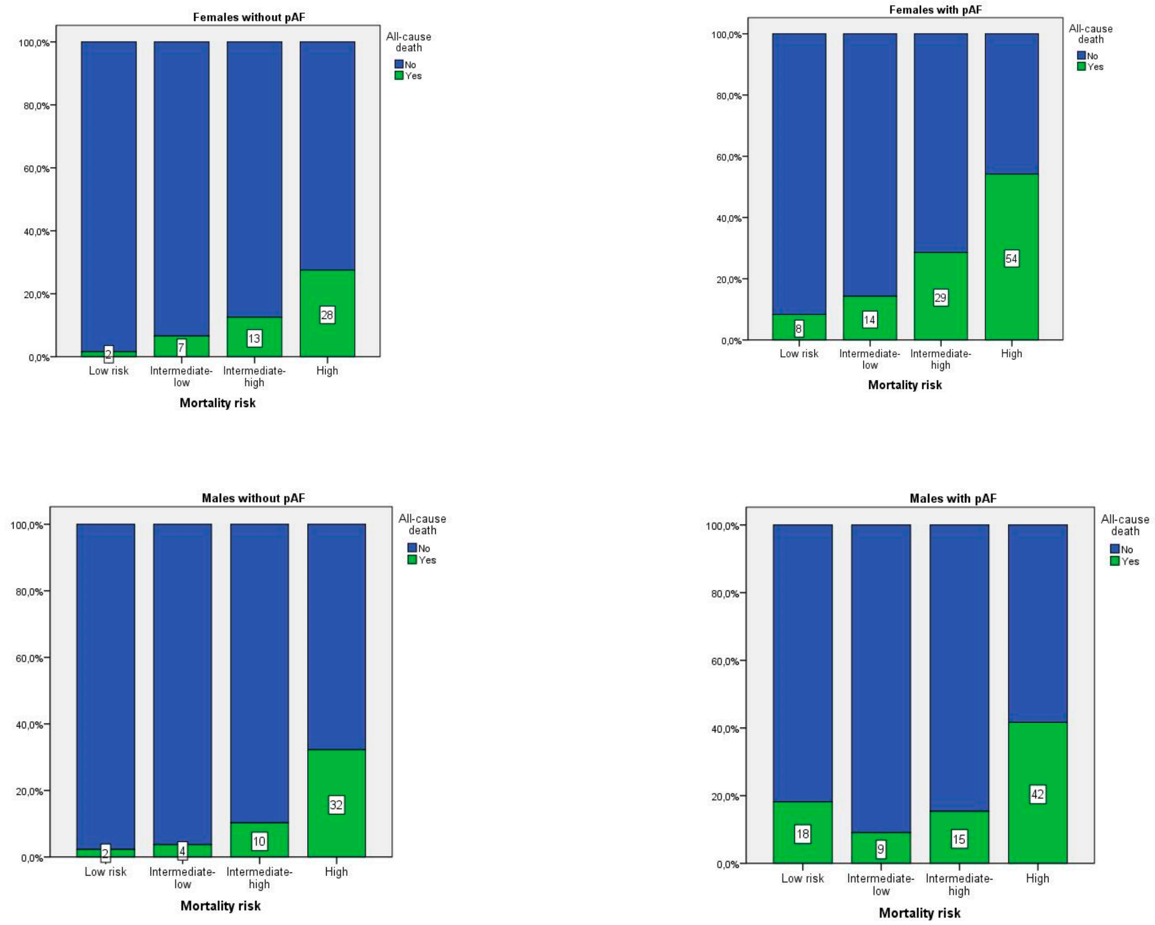

AF-atrial fibrillation
